# Avoiding lead-time bias by estimating stage-specific proportions of cancer and non-cancer deaths

**DOI:** 10.1007/s10552-023-01842-4

**Published:** 2024-01-18

**Authors:** Ellen T. Chang, Christina A. Clarke, Graham A. Colditz, Allison W. Kurian, Earl Hubbell

**Affiliations:** 1 GRAIL, LLC, 1525 O’Brien Ave, Menlo Park, CA 94025 USA; 2 Institute for Public Health and Division of Public Health Sciences, Department of Surgery, Washington University School of Medicine, St. Louis, MO USA; 3 Division of Oncology, Department of Medicine, and Department of Epidemiology & Population Health, Stanford School of Medicine, Stanford, CA USA

**Keywords:** Cancer, Early detection of cancer, Cause of death, Cancer mortality

## Abstract

**Purpose:**

Understanding how stage at cancer diagnosis influences cause of death, an endpoint that is not susceptible to lead-time bias, can inform population-level outcomes of cancer screening.

**Methods:**

Using data from 17 US Surveillance, Epidemiology, and End Results registries for 1,154,515 persons aged 50–84 years at cancer diagnosis in 2006–2010, we evaluated proportional causes of death by cancer type and uniformly classified stage, following or extrapolating all patients until death through 2020.

**Results:**

Most cancer patients diagnosed at stages I–II did not go on to die from their index cancer, whereas most patients diagnosed at stage IV did. For patients diagnosed with any cancer at stages I–II, an estimated 26% of deaths were due to the index cancer, 63% due to non-cancer causes, and 12% due to a subsequent primary (non-index) cancer. In contrast, for patients diagnosed with any stage IV cancer, 85% of deaths were attributed to the index cancer, with 13% non-cancer and 2% non-index-cancer deaths. Index cancer mortality from stages I–II cancer was proportionally lowest for thyroid, melanoma, uterus, prostate, and breast, and highest for pancreas, liver, esophagus, lung, and stomach.

**Conclusion:**

Across all cancer types, the percentage of patients who went on to die from their cancer was over three times greater when the cancer was diagnosed at stage IV than stages I–II. As mortality patterns are not influenced by lead-time bias, these data suggest that earlier detection is likely to improve outcomes across cancer types, including those currently unscreened.

**Supplementary Information:**

The online version contains supplementary material available at 10.1007/s10552-023-01842-4.

## Introduction

Evaluating the potential impact of cancer screening on the population burden of cancer can be complicated by lead-time bias. By extending survival time from earlier diagnosis without affecting lifespan, lead-time bias can invalidate analyses of survival as an endpoint [1]. Analyses of mortality as an endpoint, in contrast, can overcome lead-time bias by incorporating long-term follow-up until death for all patients. In particular, identifying differences in causes of death by stage at cancer diagnosis can shed light on cancer types with the greatest potential for benefit from earlier detection, such as types with a high proportion of deaths when diagnosed at late stages, but not early stages. This has particular value with the advent of multi-cancer early detection (MCED) tests, which can potentially be used to screen concurrently for dozens of cancer types that currently lack other screening modalities [2].

Quantifying cause-specific mortality by stage at diagnosis also clarifies whether earlier detection of individual or multiple cancer types is likely to have a statistically observable impact on all-cause mortality, which is often identified as a primary or secondary outcome of interest in cancer screening trials. In addition, understanding the causes of death among cancer patients by stage at diagnosis can inform disease management, including prioritization of secondary prevention strategies, such as screening for second cancers and other chronic diseases.

Previous studies of causes of death among cancer patients have typically focused on one or a few cancer types [3–7] or specific causes of death [8, 9]. A prior study of causes of death across all cancer types did not report results by stage at diagnosis [10]. Therefore, to gain greater insight into mortality patterns by stage of cancer at diagnosis, while using long-term mortality data to measure the population-level impact of earlier-stage cancer diagnosis without lead-time bias, we undertook a novel analysis of causes of death by type and stage among US cancer patients using population-based cancer registry data.

## Materials and methods

We obtained cancer incidence and survival data for this study from the US Surveillance, Epidemiology, and End Results (SEER) population-based cancer registries for 17 geographic regions including diagnoses from 2006 to 2010, with follow-up for mortality through December 31, 2020 [11]. These diagnosis years were selected to enable uniform classification of cancer stage according to the 6th edition of the American Joint Committee on Cancer (AJCC) staging manual [12], and to provide at least 10 years (up to 14 years and 11 months) of follow-up after cancer diagnosis.

We included all patients diagnosed with a first incident cancer (hereafter referred to as the index cancer) at ages 50–84 years, excluding those with missing age data. Cases younger than 50 years at diagnosis were excluded due to relatively low general-population mortality, corresponding to a high degree of censorship (i.e., survival past the end of follow-up). Cases were grouped by primary anatomic site using topography codes from the International Classification of Diseases for Oncology, 3rd edition (ICD-O-3), and by AJCC stage. Those with unknown or missing stage were grouped separately; this group included patients with primary brain/nervous system cancer, myeloma, or leukemia, because these types lack AJCC 6th edition staging criteria. We separately classified breast cancer as hormone receptor (HR)-positive, HR-negative, or HR-unknown using SEER Extent of Disease codes for estrogen receptor and progesterone receptor status, and lung cancer as small-cell or non-small-cell carcinoma using ICD-O-3 morphology codes.

Deaths recorded by SEER were classified based on death certificates as being due to the index cancer, a non-index cancer (i.e., a subsequent primary cancer other than the index cancer), or non-cancer causes (i.e., conditions other than cancer). Information on cancer stage at death was not available. We excluded subjects known to be deceased but with a missing or unknown cause of death (0.8%). SEER did not classify any patients as having died from a second primary cancer (including contralateral cancer) at the same anatomic site as the index cancer. Among the 26 standard non-cancer causes of death classified by SEER, we combined tuberculosis, syphilis, and other infectious/parasitic diseases as “other infectious diseases” (apart from septicemia, which was classified separately); hypertension without heart disease, atherosclerosis, aortic aneurysm/dissection, and other diseases of arteries/arterioles/capillaries as “other circulatory diseases” (apart from heart disease and cerebrovascular disease, each of which was classified separately); accidents/adverse events and homicide/legal intervention as “accidents/external causes of death” (apart from suicide/self-injury, which was classified separately); and in situ/benign/unknown-behavior neoplasms, stomach/duodenal ulcers, complications of pregnancy/childbirth/puerperium, congenital anomalies, certain conditions originating in the perinatal period, symptoms/signs/ill-defined conditions, and other causes of death as “other.”

Because some patients survived to their last contact date (60% of those diagnosed with stages I–II cancer, 32% of those diagnosed at stage III, and 9% of those diagnosed at stage IV; Table 1), and the proportion of survivors differed systematically by cancer stage and type, we extrapolated the cause of death for all subjects without observed death. This extrapolation minimized selection bias that otherwise would have occurred due to systematic differences in the probability of observing deaths from index cancers (which typically occur relatively soon after diagnosis) and deaths from other causes (which are more likely to occur later, often beyond five years after diagnosis). We extrapolated the likely cause of death in two ways (Online Resource F1). First, for patients who were lost to follow-up before the maximum time, we allocated causes of death based on the observed distribution of causes of death in the corresponding year of follow-up after the index cancer diagnosis. Second, for patients who were still alive at the end of study follow-up, we allocated causes of death based on the observed distribution of causes of death in the final four years of follow-up, without explicitly modeling future mortality dates. This extrapolation is supported by the observed plateau in risk of index cancer death approximately 10 years after diagnosis, generally equating to statistical “cure” [13]. Thus, the entire analytic cohort was followed until death by observation or extrapolation. Our rationale for not studying a cohort of patients diagnosed in earlier years—which would have a larger proportion of observed deaths—was to prioritize data incorporating more current cancer staging and treatment practices. To illustrate the roles of imputation and extrapolation by stage at diagnosis, Online Resource F2 shows the stage-specific distributions of causes of death overall and by computational step, including observation (for subjects who died during follow-up), imputation (for subjects lost to follow-up), or extrapolation (for subjects alive at the end of follow-up).

**Table 1:** Characteristics of cancer cases of all types combined by stage at diagnosis, ages 50–84 years at diagnosis from 2006 to 2010, followed for mortality through 2020, Surveillance, Epidemiology, and End Results (SEER) 17 registries

		Stage I		Stage II		Stage III		Stage IV		Unknown/Missing Stage	
		*n*	%	*n*	%	*n*	%	*n*	%	*n*	%
Vital Status at End of Observed Follow-Up											
	Alive	161,499	60	194,520	60	48,758	32	19,072	9	55,778	27
	Dead	107,341	40	131,503	40	103,718	68	183,511	91	148,815	73
Age at Diagnosis (Years)											
	50–54	39,394	15	35,488	11	20,062	13	22,935	11	22,286	11
	55–59	44,506	17	51,335	16	24,922	16	30,449	15	26,917	13
	60–64	46,741	17	62,202	19	26,903	18	34,794	17	30,354	15
	65–69	44,344	16	62,675	19	26,029	17	33,721	17	31,360	15
	70–74	37,319	14	50,958	16	21,680	14	30,514	15	31,218	15
	75–79	32,165	12	39,018	12	18,707	12	28,093	14	31,855	16
	80–84	24,371	9	24,347	7	14,173	9	22,077	11	30,603	15
Sex											
	Male	93,615	35	237,889	73	77,464	51	115,480	57	112,247	55
	Female	175,225	65	88,134	27	75,012	49	87,103	43	92,346	45
Race/Ethnicity											
	White, Non-Hispanic	207,156	77	234,134	72	110,363	72	145,710	72	145,923	71
	Black, Non-Hispanic	19,332	7	39,326	12	15,891	10	22,910	11	20,385	10
	Hispanic	21,857	8	29,864	9	14,250	9	18,739	9	21,559	11
	Asian American/Pacific Islander, Non-Hispanic	17,218	6	18,741	6	10,767	7	13,724	7	12,992	6
	American Indian/Alaska Native, Non-Hispanic	1,298	0	1,464	0	936	1	1,255	1	1,184	1
	Other/Unknown, Non-Hispanic	1,979	1	2,494	1	269	0	245	0	2,550	1
Observed Follow-Up Duration (Months)											
	0–12	18,626	7	20,504	6	38,697	25	121,495	60	72,190	35
	13–24	11,065	4	13,526	4	17,693	12	25,841	13	18,479	9
	25–36	9,666	4	11,276	3	10,520	7	11,849	6	11,517	6
	37–48	8,565	3	10,275	3	7,552	5	6,906	3	8,409	4
	49–60	8,211	3	9,906	3	5,734	4	4,552	2	7,008	3
	61–72	8,092	3	9,771	3	4,851	3	3,347	2	6,235	3
	73–84	8,198	3	9,881	3	4,222	3	2,608	1	5,637	3
	85–96	8,226	3	9,830	3	3,797	2	2,215	1	5,115	3
	97–108	8,297	3	10,215	3	3,535	2	1,955	1	4,819	2
	109–120	11,276	4	13,889	4	4,260	3	2,178	1	5,545	3
	121–132	42,691	16	50,082	15	13,569	9	5,752	3	16,042	8
	133–144	38,464	14	46,579	14	12,297	8	4,537	2	14,072	7
	145–156	34,076	13	41,522	13	10,114	7	3,832	2	11,880	6
	157–168	29,892	11	38,862	12	8,862	6	3,235	2	10,002	5
	169–179	23,495	9	29,905	9	6,773	4	2,281	1	7,643	4
Index Cancer Type											
	Bladder	10,441	4	4,846	1	2,065	1	3,511	2	1,856	1
	Breast, All	75,417	27	49,486	15	17,295	10	8,482	4	7,839	4
	Breast, Hormone-Receptor-Positive	63,106	23	37,634	12	12,314	7	5,498	2	3,912	2
	Breast, Hormone-Receptor-Negative	9,484	3	9,818	3	4,250	3	1,798	1	1,001	0
	Brain/Other Nervous System	0	0	0	0	0	0	0	0	12,403	6
	Cervix	2,228	1	1,060	0	1,432	1	1,167	1	609	0
	Colon/Rectum	25,571	9	25,550	8	26,014	16	20,516	9	9,358	5
	Esophagus	1,861	1	2,011	1	2,201	1	4,104	2	1,771	1
	Kidney	19,568	7	3,237	1	5,311	3	6,589	3	2,298	1
	Larynx	3,275	1	1,454	0	1,448	1	2,544	1	732	0
	Leukemia	0	0	0	0	0	0	0	0	27,369	13
	Liver/Intrahepatic Bile Duct	6,257	2	3,474	1	4,351	3	3,785	2	5,076	2
	Lung, All	26,725	10	6,501	2	36,810	22	70,601	32	15,565	8
	Lung, Non-Small-Cell	21,048	8	4,805	2	21,707	15	35,530	18	5,155	4
	Lung, Small-Cell	846	0	381	0	5,896	4	13,020	7	1,359	1
	Lymphoma	12,698	5	7,333	2	8,206	6	16,721	9	4,014	3
	Melanoma	29,446	11	5,051	2	2,632	2	1,732	1	4,351	3
	Myeloma	7	0	0	0	0	0	0	0	16,528	11
	Oral Cavity/Pharynx	4,265	2	2,837	1	3,753	3	11,339	6	4,782	3
	Ovary	2,793	1	1,193	0	5,892	4	4,737	2	1,886	1
	Pancreas	1,829	1	6,942	2	2,531	2	15,281	8	4,357	3
	Prostate	202	0	191,580	60	16,717	11	13,032	7	15,635	11
	Stomach	3,958	2	1,880	1	1,848	1	6,727	3	3,722	3
	Thyroid	9,708	4	2,392	1	3,655	2	2,591	1	1,490	1
	Uterus	24,017	9	2,456	1	4,653	3	2,414	1	3,949	3
	Other Types	8,574	3	6,740	2	5,662	4	6,710	3	59,003	41

To estimate the change in the distribution of causes of death that could arise from earlier stage at diagnosis due to universal cancer screening (e.g., with an MCED test), we calculated proportions of cause-specific deaths under two hypothetical scenarios: (1) if all stage IV cancers were shifted to stage III, and (2) if all stage IV cancers were equally distributed among stages I, II, and III [14]. We calculated these values separately for each index cancer type, and then summated them across all cancer types.

Analyses were conducted using SEER*Stat version 8.4.1 [15] and the R statistical programming language, including the tidyverse package [16, 17]. This study was not subject to institutional review board approval or informed consent due to its secondary use of de-identified data. Code and data are available at https://github.com/grailbio-publications/Chang_Causes_of_Death. Due to privacy concerns related to providing data with small numbers of events in some cells, we provide the specifications for the original SEER data draw, along with synthetic data generated to match the large-scale statistics to demonstrate the code. Figures and tables reported in this paper are from the original data only. Interested individuals can retrieve the original SEER data from the draw specifications.

## Results

The characteristics of all 1,154,515 first primary cancer cases by stage at diagnosis, including vital status at the end of observed (not extrapolated) follow-up through 2020, age at diagnosis, sex, race/ethnicity, duration of observed follow-up, and index cancer type, are shown in Table 1. The five most common incident index cancer types included in the analysis were prostate (*n* = 237,166), breast (*n* = 158,519), lung (*n* = 156,202), colon/rectum (*n* = 107,009), and lymphoma (*n* = 48,972).

**Fig. 1:**
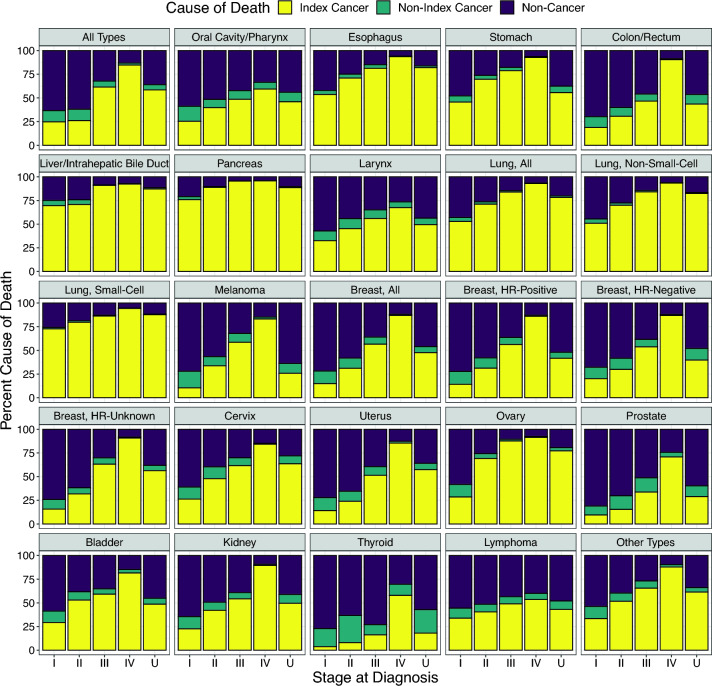
Distribution of causes of death (extrapolated if not observed) by stage at diagnosis for cancer cases overall and by first primary incident cancer type, ages 50–84 years at diagnosis from 2006 to 2010, followed for mortality through 2020, Surveillance, Epidemiology, and End Results (SEER) 17 registries. Cancer types are ordered by topography code according to the International Classification of Diseases for Oncology, 3rd Edition. *HR* hormone receptor, *U* unknown/missing stage

After extrapolation, the five most common causes of index cancer death were lung (*n* = 127,470, including 69,769 non-small cell and 19,486 small cell), prostate (*n* = 49,120), breast (*n* = 47,604, including 34,010 HR-positive and 9,095 HR-negative), colon/rectum (*n* = 47,427), and pancreas (*n* = 28,471). Underlying data are provided in Table 2. Figure 1 shows the extrapolated proportions of deaths due to index cancers, non-index cancers, or non-cancer causes for all cancer types combined and for each index cancer type. The distribution of causes of death among cases with unknown or missing stage at index cancer diagnosis generally resembled that for cases diagnosed at stage III.

**Table 2:** Stage-specific distribution of causes of death (extrapolated if not observed) among cancer cases by index cancer type, ages 50–84 years at diagnosis from 2006 to 2010, followed for mortality through 2020, Surveillance, Epidemiology, and End Results (SEER) 17 registries

		Cause of Death					
		Index Cancer		Non-Index Cancer		Non-Cancer	
Index Cancer	Stage at Diagnosis	*n*	%	*n*	%	*n*	%
All Types							
	I	66,737	25	31,785	12	170,318	63
	II	85,179	26	39,125	12	201,719	62
	III	93,826	62	9,468	6	49,183	32
	IV	171,607	85	4,279	2	26,698	13
	Unknown/Missing	119,567	58	11,375	6	73,651	36
Bladder							
	I	3,060	29	1,251	12	6,129	59
	II	2,567	53	428	9	1,850	38
	III	1,224	59	116	6	725	35
	IV	2,867	82	126	4	518	15
	Unknown/Missing	903	49	115	6	837	45
Breast, All							
	I	11,259	15	10,032	13	54,126	72
	II	15,448	31	5,289	11	28,748	58
	III	9,788	57	1,281	7	6,227	36
	IV	7,377	87	94	1	1,011	12
	Unknown/Missing	3,733	48	509	6	3,597	46
Breast, HR-positive							
	I	8,929	14	8,618	14	45,559	72
	II	11,783	31	4,049	11	21,802	58
	III	6,935	56	911	7	4,468	36
	IV	4,728	86	64	1	706	13
	Unknown/Missing	1,635	42	248	6	2,030	52
Breast, HR-negative							
	I	1,906	20	1,156	12	6,422	68
	II	2,944	30	1,153	12	5,722	58
	III	2,284	54	340	8	1,626	38
	IV	1,562	87	21	1	215	12
	Unknown/Missing	399	40	123	12	479	48
Breast, HR-unknown							
	I	420	16	268	10	2,008	74
	II	613	32	126	6	1,206	62
	III	435	64	49	7	201	29
	IV	1,051	91	11	1	95	8
	Unknown/Missing	1,646	56	166	6	1,102	38
Cervix							
	I	586	26	284	13	1,358	61
	II	507	48	133	13	420	40
	III	883	62	120	8	429	30
	IV	984	84	10	1	173	15
	Unknown/Missing	388	64	51	8	171	28
Colon/Rectum							
	I	4,806	19	2,896	11	17,869	70
	II	7,853	31	2,332	9	15,364	60
	III	12,134	47	1,952	8	11,928	46
	IV	18,555	90	206	1	1,755	9
	Unknown/Missing	4,078	44	950	10	4,330	46
Esophagus							
	I	999	54	78	4	784	42
	II	1,429	71	82	4	500	25
	III	1,789	81	84	4	328	15
	IV	3,841	94	22	1	240	6
	Unknown/Missing	1,452	82	33	2	286	16
Kidney							
	I	4,428	23	2,518	13	12,622	65
	II	1,363	42	279	9	1,595	49
	III	2,881	54	352	7	2,078	39
	IV	5,901	90	79	1	608	9
	Unknown/Missing	1,140	50	214	9	944	41
Larynx							
	I	1,065	33	337	10	1,872	57
	II	659	45	156	11	640	44
	III	812	56	131	9	505	35
	IV	1,718	68	153	6	673	26
	Unknown/Missing	362	49	51	7	319	44
Liver/Intrahepatic Bile Duct							
	I	4,353	70	345	6	1,559	25
	II	2,462	71	172	5	840	24
	III	3,958	91	20	0	373	9
	IV	3,497	92	16	0	272	7
	Unknown/Missing	4,427	87	72	1	577	11
Lung, All							
	I	14,134	53	1,115	4	11,476	43
	II	4,626	71	158	2	1,717	26
	III	30,907	84	482	1	5,420	15
	IV	65,620	93	377	1	4,604	7
	Unknown/Missing	12,182	78	238	2	3,145	20
Lung, Non-Small-Cell							
	I	10,742	51	953	5	9,353	44
	II	3,363	70	119	2	1,323	28
	III	18,255	84	329	2	3,123	14
	IV	33,160	93	204	1	2,166	6
	Unknown/Missing	4,249	82	54	1	852	17
Lung, Small-Cell							
	I	615	73	13	2	217	26
	II	304	80	6	2	71	19
	III	5,091	86	46	1	759	13
	IV	12,285	94	34	0	701	5
	Unknown/Missing	1,191	88	10	1	158	12
Lymphoma							
	I	4,305	34	1,334	11	7z,059	56
	II	2,963	40	602	8	3,768	51
	III	4,020	49	613	7	3,574	44
	IV	8,958	54	1,091	7	6,672	40
	Unknown/Missing	1,732	43	358	9	1,924	48
Melanoma							
	I	3,101	11	5,151	17	21,193	72
	II	1,706	34	489	10	2,856	57
	III	1,544	59	244	9	843	32
	IV	1,442	83	33	2	257	15
	Unknown/Missing	1,126	26	450	10	2,776	64
Oral Cavity/Pharynx							
	I	1,086	25	671	16	2,508	59
	II	1,128	40	249	9	1,460	51
	III	1,826	49	339	9	1,588	42
	IV	6,736	59	803	7	3,801	34
	Unknown/Missing	2,201	46	481	10	2,100	44
Ovary							
	I	799	29	368	13	1,626	58
	II	826	69	61	5	307	26
	III	5,170	88	105	2	617	10
	IV	4,333	91	47	1	357	8
	Unknown/Missing	1,457	77	64	3	365	19
Pancreas							
	I	1,389	76	57	3	383	21
	II	6,175	89	55	1	712	10
	III	2,415	95	7	0	109	4
	IV	14,634	96	42	0	605	4
	Unknown/Missing	3,858	89	55	1	444	10
Prostate							
	I	19	10	19	9	164	81
	II	29,688	15	27,419	14	134,472	70
	III	5,637	34	2,521	15	8,560	51
	IV	9,236	71	627	5	3,169	24
	Unknown/Missing	4,540	29	1,766	11	9,328	60
Stomach							
	I	1,807	46	256	6	1,896	48
	II	1,312	70	77	4	491	26
	III	1,456	79	66	4	326	18
	IV	6,241	93	62	1	424	6
	Unknown/Missing	2,074	56	253	7	1,395	37
Thyroid							
	I	363	4	1,857	19	7,488	77
	II	192	8	684	29	1,516	63
	III	593	16	394	11	2,668	73
	IV	1,503	58	304	12	784	30
	Unknown/Missing	269	18	370	25	851	57
Uterus							
	I	3,407	14	3,291	14	17,318	72
	II	591	24	258	10	1,607	65
	III	2,394	51	424	9	1,836	39
	IV	2,062	85	47	2	306	13
	Unknown/Missing	2,265	57	266	7	1,417	36
Other Types							
	I	2,872	33	1,095	13	4,614	54
	II	3,490	52	576	9	2,674	40
	III	3,710	66	432	8	1,519	27
	IV	5,899	88	173	3	638	10
	Unknown/Missing	70,888	61	5,323	5	39,092	34

Row percentages may not sum to 100% due to rounding error

*HR* hormone receptor

Across all cancer types, the majority of deaths for cancer patients diagnosed at stages I and II were due to causes other than the index cancer. For stage I cancer of all types, 63% of deaths were due to non-cancer causes, 25% were due to the index cancer, and 12% were due to a subsequent primary non-index cancer; that is, 75% of deaths among patients with stage I cancer were not attributable to the index cancer (Fig. 1). Similarly, at stage II, 74% of deaths were not due to the index cancer, including 62% due to non-cancer causes and 12% due to a non-index cancer. At stage III, the majority of deaths (62%) were due to the index cancer, with 32% due to non-cancer causes and 6% due to a non-index cancer. As expected, the highest proportion of deaths from the index cancer (85%) occurred at stage IV, where 13% of deaths were due to non-cancer causes and 2% were due to a non-index cancer. From another perspective, of the 417,348 index cancer deaths with known stage at diagnosis, 41% were diagnosed at stage IV, 22% at stage III, 20% at stage II, and 16% at stage I.

These proportions were not appreciably affected after excluding index cancers with currently recommended screening protocols in the US (i.e., colorectal, breast, lung, and cervix [18]). For the remaining unscreened cancers diagnosed at stages I–II, 63% of deaths were due to non-cancer causes, 24% due to the index cancer, and 13% due to a non-index cancer. Additional exclusion of prostate cancer as a screened cancer did not change the distribution of causes of death at stage I, but doubled the percentage of deaths due to the index cancer at stage II (52%), with corresponding decreases in non-cancer deaths (41%) and non-index cancer deaths (7%) after stage II index cancer.

We estimated that 33,958 (6%) fewer deaths from index cancers would occur if, in theory, universal cancer screening were implemented in this population such that all of the stage IV index cancers were instead detected at stage III. If universal cancer screening instead led to detection of one third of the stage IV index cancers at each of stages I, II, and III, then 62,092 (12%) fewer deaths from index cancers would theoretically occur.

The pattern of a lower proportion of index cancer deaths at earlier stages was observed across all index cancer types, but absolute percentages varied substantially by type (Fig. 1). The lowest proportions of deaths from early-stage index cancers were seen for thyroid (5% of deaths due to the index cancer at stages I–II), melanoma (14%), uterus (15%), prostate (15%), and breast (21% overall and HR-positive). In contrast, the highest proportions of deaths from early-stage index cancers were observed for cancers of the pancreas (86% of deaths due to the index cancer at stages I-II), liver/intrahepatic bile duct (70%), esophagus (63%), lung (56% overall, 75% small-cell, 55% non-small-cell), and stomach (53%).

### Non-index cancer deaths

Among stage I index cancer cases, the types with the highest proportion of deaths due to a subsequent primary non-index cancer were thyroid (19% of deaths due to another cancer), melanoma (17%), oral cavity/pharynx (16%), uterus (14%), and breast, cervix, kidney, and ovary (all 13%) (Fig. 1). These percentages reflect a combination of relatively young average age at diagnosis and low early-stage mortality for the index cancer, and possibly shared risk factors between index and non-index cancers.

**Fig. 2:**
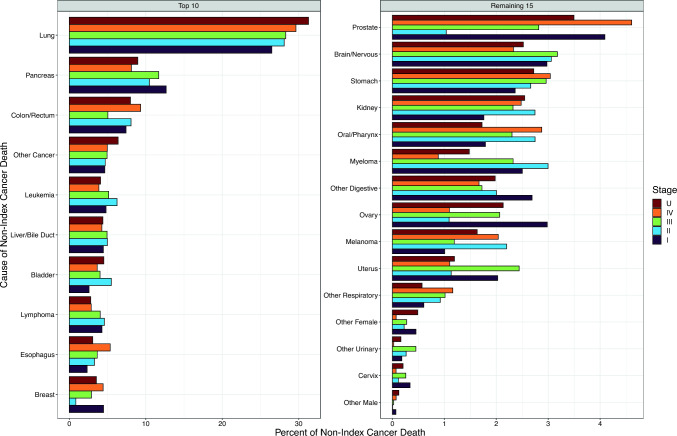
Distribution of detailed non-index cancer causes of death (extrapolated if not observed) by stage at diagnosis for cancer cases of all types combined, ages 50–84 years at diagnosis from 2006 to 2010, followed for mortality through 2020, Surveillance, Epidemiology, and End Results (SEER) 17 registries. Leading causes of non-index cancer death are ordered by frequency for stage I index cancer, with different scales for left and right panels. *U* unknown/missing stage

Figure 2 illustrates the stage-specific proportion of deaths by detailed non-index cancer type among the 96,031 cancer cases (8% of all cases) who died from a subsequent non-index cancer (data in Online Resource T1). Online Resource F3 shows these distributions for the most common index cancer types in this analysis, i.e., breast (*n* = 17,205 non-index cancer deaths), colon/rectum (*n* = 8,336), lung (*n* = 2,370), and prostate (*n* = 32,353). For all cancer types combined, the leading non-index cancer cause of death was lung cancer, with little variation in the percentage of attributed deaths across stages I–IV index cancers (27%–30% of non-index-cancer deaths within stage; 0.6%–3% of total deaths within stage) (Fig. 2). The next most common non-index cancer causes of death were pancreatic cancer (8%–13% of stage-specific non-index-cancer deaths), colorectal cancer (5%–9%), leukemia (4%–6%), and liver/intrahepatic bile duct cancer (4%–5%). Except for breast and prostate cancers, which were largely precluded from being common causes of non-index cancer death in part by their high frequency as index cancers, the leading types of non-index cancer death generally matched the most common causes of cancer death in the US population [19].

The patterns of non-index cancer deaths were largely mirrored in analyses by type of index cancer (Online Resource F3; data not shown for other index cancer types). That is, lung cancer generally caused the plurality of non-index cancer deaths, especially for smoking-related index cancers (e.g., oral cavity/pharynx: 46%–51% of non-index cancer deaths due to lung cancer, depending on stage; bladder: 39%–50%; esophagus: 20%–52%, respectively), followed by other leading causes of cancer death in the general population. Some concordance was also evident between index cancers and deaths from non-index cancers with shared risk factors (e.g., breast and ovary).

### Non-cancer deaths

**Fig. 3:**
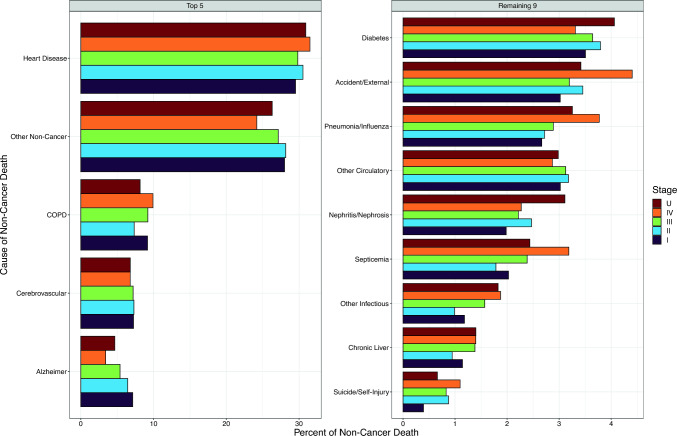
Distribution of detailed non-cancer causes of death (extrapolated if not observed) by stage at diagnosis for cancer cases of all types combined, ages 50–84 years at diagnosis from 2006 to 2010, followed for mortality through 2020, SEER 17 registries. Leading causes of non-cancer death are ordered by frequency for stage I index cancer, with different scales for left and right panels. *COPD* chronic obstructive pulmonary disease; *U* unknown/missing stage

The stage-specific distribution of detailed causes of death among the 521,570 cancer cases (45% of all cases) who died from non-cancer causes is shown in Fig. 3 (data in Online Resource T1). Online Resource F4 illustrates the corresponding distributions for cancers of the breast (*n* = 93,710 non-cancer deaths), colon/rectum (*n* = 51,246), lung (*n* = 26,362), and prostate (*n* = 155,693). Across nearly all index cancer types at all stages, heart disease was the leading cause of non-cancer death, generally accounting for 20%–40% of non-cancer deaths (1%–24% of total deaths within stage, depending on index cancer type and stage, i.e., lowest for stage IV pancreatic cancer and highest for stage I prostate cancer). Exceptions to this pattern were cancer of the liver/intrahepatic bile duct, for which “other infectious diseases” (a category that includes hepatitis B and C) was the leading cause of non-cancer death at stages I–III (26%–38% of non-cancer deaths); and lung cancer, including non-small-cell and small-cell subtypes, for which chronic obstructive pulmonary disease (COPD) was the most common cause of death at stage I (27%–29%; also at stage II for small-cell lung cancer [30%]).

After heart disease, the next most common specific cause of non-cancer death was COPD, which was responsible for 7%–10% of non-cancer deaths at each stage of all cancers combined. The percentages of non-cancer deaths attributed to COPD were highest for smoking-related index cancer types, such as lung (19%–28% of non-cancer deaths, depending on stage), bladder (11%–14%), and oral cavity/pharynx (9%–12%) (Online Resource F4; data not shown for other index cancer types). Some causes of death, such as Alzheimer disease and diabetes, were somewhat more common after stages I–II cancer than stage IV, whereas others, such as septicemia, other infectious disease, and suicide/self-inflicted injury, were slightly more frequent after stage IV than stages I–II cancer. Otherwise, the distribution of non-cancer causes of death appeared to be fairly steady across stages of index cancer, and broadly corresponded to the most common non-cancer causes of death in the general US population of older adults [20].

### Results by age, sex, and race/ethnicity

**Fig. 4:**
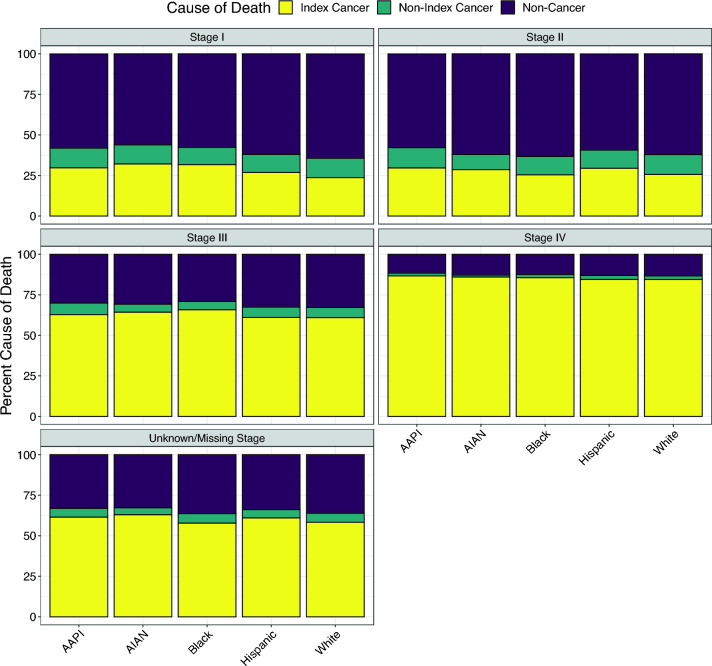
Race/ethnicity-stratified distribution of causes of death (extrapolated if not observed) by stage at diagnosis for cancer cases of all types combined, ages 50–84 years at diagnosis from 2006 to 2010, followed for mortality through 2020, Surveillance, Epidemiology, and End Results (SEER) 17 registries. “Hispanic” includes all races and does not overlap with other racial/ethnic groups. *AIAN* American Indian/Alaska Native, *AAPI* Asian American/Pacific Islander, *U* unknown/missing stage

Stratification by 5-year age group at diagnosis revealed a generally increasing proportion of non-cancer deaths, accompanied by decreasing proportions of index cancer and non-index cancer deaths, with older age at diagnosis (Online Resource F5). This pattern is most likely attributable to substantial competing non-cancer causes of death at older ages, as opposed to increased treatability of cancer. Stratification by sex (as classified by SEER) indicated no substantial differences between men and women after excluding breast cancer and sex-specific cancers (Online Resource F6). Stratification by race/ethnicity identified modestly higher proportions of deaths from stage I index cancer among all non-White groups than non-Hispanic White patients (Fig. 4; data in Online Resource T2). Whereas 24% of deaths among non-Hispanic White stage I cancer cases were attributed to the index cancer, 32% of non-Hispanic Black cases, 32% of non-Hispanic American Indian/Alaska Native (AIAN) cases, 30% of non-Hispanic Asian American/Pacific Islander (AAPI) cases, and 27% of Hispanic cases died of their stage I index cancer. The apparent racial/ethnic disparity in index cancer deaths diminished with advancing stage at diagnosis, with all groups experiencing 84%–87% of deaths from the index cancer after diagnosis at stage IV.

## Discussion

To our knowledge, this is the first study to systematically evaluate the distribution of causes of death among all major cancer types by stage at diagnosis in a representative population. Our analysis takes advantage of high-quality population-based SEER cancer registry data, which allows consideration of uniformly classified stage and other characteristics such as age and year of diagnosis, combined with nearly 15 years of follow-up. By reporting stage-specific results, we quantified the potential reduction in cause-specific and all-cause mortality through early cancer detection, which can shift late-stage cancer incidence to earlier, more curable stages. Our use of long-term mortality data in a cohort of patients followed all the way to death (by extrapolation if not observed) allowed us to avoid lead-time bias, which can otherwise threaten comparisons of survival outcomes by cancer stage.

The patterns that we observed across all cancer types combined represent the average risks of all cancer patients aged 50–84 years. This information is broadly relevant to public health because individuals cannot predict or choose which cancer type they develop. Averaged across the representative spectrum of cancer types arising in a general population, earlier stage at diagnosis translated to a threefold lower proportion of cause-specific death from cancer.

Cancers with the largest discrepancies in proportional index cancer deaths between stages IV and I at diagnosis, including neoplasms with a relatively good overall prognosis, such as uterus, breast, colon/rectum, melanoma, kidney, ovary, and prostate (all with > 60% absolute difference in index cancer deaths between stages IV and I), may yield the most visible population-level benefit in cause-specific mortality through early detection. Some of this apparent benefit is probably inflated by overdiagnosis—that is, detection of clinically insignificant indolent, early-stage cancers—making it important for screening tests and/or follow-up pathological assessments to distinguish between potentially harmful and harmless cancers. However, even cancer types with a relatively poor overall prognosis and a high proportion of stage I index cancer deaths, such as pancreas, liver/intrahepatic bile duct, esophagus, lung, and stomach, exhibited a 20%–47% absolute difference in cause-specific deaths between stages IV and I. Some mortality differences by stage may be explained in part by different biological and prognostic characteristics between cancers diagnosed at earlier and later stages, even among clinically significant (not overdiagnosed) cancers.

Given that any cancer type contributes modestly to overall mortality, single-cancer screening (even if perfect) generally cannot be expected to appreciably affect all-cause mortality [21–23]. For example, even lung cancer, the leading cause of cancer death (24% of index cancer deaths), accounted for 11% of overall deaths in our study population. Currently recommended lung cancer screening with full uptake and adherence is estimated to reduce lung cancer mortality by 13% [24], corresponding to a 3% reduction in cancer mortality and a 1% reduction in all-cause mortality in our study population. Multi-cancer screening strategies, in contrast, can potentially have a greater impact on population-wide all-cause mortality by simultaneously reducing cause-specific mortality from dozens of cancers. We estimated that shifting index cancers from stage IV to stage III with an MCED test, as an adjunct to current cancer screening, would theoretically reduce index cancer deaths by 6%, and shifting stage IV to stages I, II, and III would reduce index cancer deaths by 12% in this population. (For context, a perfect screening program that shifted all stage IV, III, and II index cancers to stage I, if added to existing cancer screening modalities, would theoretically result in 32% fewer deaths from index cancers.) Additional cancer deaths could potentially be averted by earlier detection of subsequent non-index cancers.

Overall, our findings are consistent with those of Zaorsky et al. [10], who used SEER data to examine causes of death among cancer patients by site, year, age, and time since diagnosis, but not stage. Adding information on stage at diagnosis enabled us to reveal distinct cause-specific mortality patterns that are obscured by combining all stages, which we found to have a substantial impact on patterns of death by index cancer versus non-index cancer or non-cancer causes.

We found that modestly higher percentages of stage I cancer patients in all major non-White racial/ethnic groups, including Black, Hispanic, AAPI, and AIAN cases, died from their index cancer than non-Hispanic White cases, but such gaps were not apparent for stage IV cancer. This racial/ethnic disparity suggests possible inequities in healthcare access and/or utilization for treatment and management of early-stage cancer. Differences in histopathologic subtype and tumor behavior for certain cancer types may also play a role [25–27]. Our findings indicate that delayed diagnosis and late-stage presentation are not the only explanations for well-known racial/ethnic disparities in cancer outcomes [28], and that even early-stage cancer may more often be lethal in non-White patients—consistent with, for example, higher breast cancer mortality among Black than non-Hispanic White women with ductal carcinoma in situ [29].

In our results, stage at index cancer diagnosis had little impact on the rankings and distributions of the most common types of non-cancer and non-index cancer causes of death experienced by cancer patients. The leading causes of non-cancer death (i.e., heart disease, COPD, cerebrovascular disease, Alzheimer disease, diabetes) and non-index cancer death (i.e., lung, pancreas, colon/rectum, leukemia, liver/intrahepatic bile duct, not including breast and prostate cancers, which were the leading index cancers) were generally the same for stages I–IV cancer survivors as they were for the total US population of older adults [19, 20].

Our study is strengthened by the high validity and completeness [30], long follow-up, and generalizable, population-based nature of the SEER data. The population covered by the SEER 17 geographic regions is socioeconomically comparable to the general US population, but has a higher proportion of Hispanic, AAPI, AIAN, other-race, and foreign-born persons [31]. Like other studies that use death certificates, ours is limited by potential misclassification of causes of death, which may vary by demographic characteristics. Differential misclassification of cause of death by stage might occur if, for instance, deaths occurring after more recent cancer diagnoses were more likely to be attributed to those cancers, regardless of whether they actually played a causal role. One of the main limitations of our study is that, due to limited follow-up time, causes of death were not observed for a large proportion of the patient cohort, especially those with early-stage cancer. We chose not to include pre-2006 cases, who would have had longer follow-up time for observed death, due to changes in cancer screening, treatment, staging, and other aspects that make earlier cases less relevant to the present. For instance, a cohort of patients aged ≥ 50 years followed completely until death as of 2020 would have had to be diagnosed in approximately 1970 or earlier. Thus, to limit selection bias that otherwise would have occurred from excluding patients without observed death, we extrapolated causes of death based on the last four years of observed data for cases still alive at the end of follow-up. Conversely, by excluding cases diagnosed after 2010, we reduced the proportion of patients with unobserved causes of death, but omitted years covering more recent advances in cancer management.

Due to the proportional mortality design of this study, we could not determine whether higher percentages of deaths from a given cause were due to an increased risk of that cause or decreased risk of an alternative cause. Also due to the proportional mortality design, our results cannot be interpreted as providing estimates of absolute or relative risk of cause-specific mortality. Because we extrapolated some deaths among cancer patients, we did not calculate standardized mortality ratios comparing cause-specific mortality risk with the general US population; however, even based on observed deaths, the risk of most specific causes of death was higher among cancer patients at every stage than in the general population, adjusting for age, sex, and race (data not shown). The purpose of this study was not to conduct a competing risks analysis, which can yield results that are more interpretable on an absolute basis, but are less readily compared on a relative basis [32] and are susceptible to lead-time bias. Finally, we did not address any issues related to changes in life-years leading to mortality events, but instead evaluated only final causes of death. Treatment at early stages may extend life, even if death eventually occurs from the index cancer, especially in younger individuals with fewer competing risks.

In conclusion, we showed that most cancer patients diagnosed at stages I–II do not go on to die of their disease, whereas most stage IV cancer is lethal. These findings, which are resistant to lead-time bias, indicate that earlier stage at diagnosis generally translates to a considerable reduction in risk of cause-specific death from cancer. Thus, earlier cancer detection across the representative spectrum of cancer types that develop in a general population has the potential to improve long-term mortality outcomes.

### Supplementary Information

Below is the link to the electronic supplementary material.

Supplementary file1 (EPS 36 kb)
Online Resource F1. Schematic of extrapolation of causes of death for subjects without observed death during follow-up. A) Original data with observed vital status at the end of follow-up, including subjects lost to follow-up. B) Imputation of causes of death for subjects lost to follow-up, based on the appropriate distribution of causes of death in each year after diagnosis. C) Observed distribution of causes of death by follow-up year after diagnosis. D) Extrapolation of future causes of death for subjects alive at the end of follow-up, based on distribution of causes of death in the last four years of follow-up.

Supplementary file2 (EPS 56 kb)
Online Resource F2. Distribution of causes of death by stage at diagnosis, overall (“final estimate”) and by computational step, including observation (“known deaths”), imputation due to loss to follow-up (“lost imputed deaths”), or extrapolation due to survival beyond the end of follow-up (“extrapolated deaths”). As shown, at stages I and II, 40% of causes of death were known from observation, 5% were imputed, and 55% were extrapolated; at stage III, 68% of causes of death were known from observation, 3% were imputed, and 29% were extrapolated; and at stage IV, 91% of causes of death were known from observation, 2% were imputed, and 8% were extrapolated. Index cancer deaths are likely to occur relatively soon after diagnosis, whereas other deaths are likely to occur later (often beyond 5 years after diagnosis). As shown, the time dependency in observed causes of death differs by stage at diagnosis, and is accounted for through extrapolation.

Supplementary file3 (EPS 55 kb)
Online Resource F3. Distribution of detailed non-index cancer causes of death (extrapolated if not observed) by stage at diagnosis for cases with primary index cancer of the breast, colon/rectum, lung, and prostate, ages 50–84 years at diagnosis from 2006–2010, followed for mortality through 2020, Surveillance, Epidemiology, and End Results (SEER) 17 registries. U: unknown/missing stage.

Supplementary file4 (EPS 44 kb)
Online Resource F4. Distribution of detailed non-cancer causes of death (extrapolated if not observed) by stage at diagnosis for cases with primary index cancer of the breast, colon/rectum, lung, and prostate, ages 50–84 years at diagnosis from 2006–2010, followed for mortality through 2020, Surveillance, Epidemiology, and End Results (SEER) 17 registries. COPD: chronic obstructive pulmonary disease; U: unknown/missing stage.

Supplementary file5 (EPS 27 kb)
Online Resource F5. Age-stratified distribution of causes of death (extrapolated if not observed) by stage at diagnosis for cancer cases of all types combined, ages 50–84 years at diagnosis from 2006–2010, followed for mortality through 2020, Surveillance, Epidemiology, and End Results (SEER) 17 registries. U: unknown/missing stage.

Supplementary file6 (EPS 13 kb)
Online Resource F6. Sex-stratified distribution of causes of death (extrapolated if not observed) by stage at diagnosis for cancer cases of all types combined, excluding breast cancer, female genital cancers, and male genital cancers, ages 50–84 years at diagnosis from 2006–2010, followed for mortality through 2020, Surveillance, Epidemiology, and End Results (SEER) 17 registries. U: unknown/missing stage.

Supplementary file7 (DOCX 23 kb)

## Data Availability

Code and data are available at https://github.com/grailbio-publications/Chang_Causes_of_Death. Due to privacy concerns related to providing data with small numbers of events in some cells, we provide the specifications for the original SEER data draw, along with synthetic data generated to match the large-scale statistics to demonstrate the code. Figures and tables reported in this paper are from the original data only. Interested individuals can retrieve the original SEER data from the draw specifications.
