# Supplementary material for: Avoiding lead-time bias by estimating stage-specific proportions of cancer and non-cancer deaths
Source: Cancer Causes Control. 2024 Jan 18;35(5):849–64. doi: 10.1007/s10552-023-01842-4 (PMC11045653; doi:10.1007/s10552-023-01842-4)
Supplement: Supplementary file 1 — Supplementary file1 (EPS 36 kb) Online Resource F1. Schematic of extrapolation of causes of death for subjects without observed death during follow-up. A) Original data with observed vital status at the end of follow-up, including subjects lost to follow-up. B) Imputation of causes of death for subjects lost to follow-up, based on the appropriate distribution of causes of death in each year after diagnosis. C) Observed distribution of causes of death by follow-up year after diagnosis. D) Extrapolation of future causes of death for subjects alive at the end of follow-up, based on distribution of causes of death in the last four years of follow-up. [file 10552_2023_1842_MOESM1_ESM.pdf]

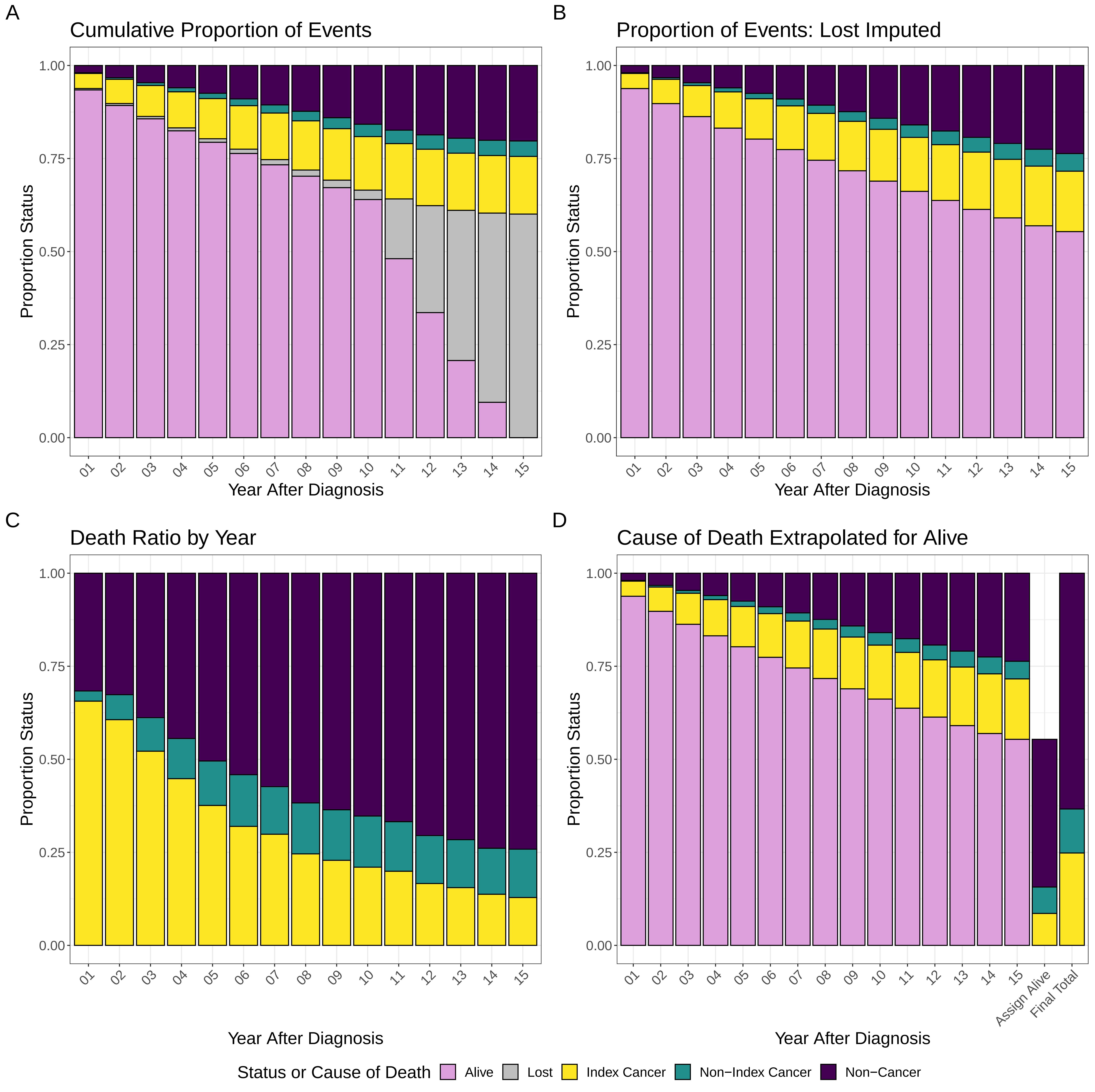
A
Cumulative Proportion of Events
B
Proportion of Events: Lost Imputed
C
Death Ratio by Year
D
Cause of Death Extrapolated for Alive
Proportion Status
Year After Diagnosis
0.00
0.25
0.50
0.75
1.00
01 02 03 04 05 06 07 08 09 10 11 12 13 14 15
Assign Alive
Final Total
Status or Cause of Death
Alive
Lost
Index Cancer
Non−Index Cancer
Non−Cancer
