# Supplementary material for: Avoiding lead-time bias by estimating stage-specific proportions of cancer and non-cancer deaths
Source: Cancer Causes Control. 2024 Jan 18;35(5):849–64. doi: 10.1007/s10552-023-01842-4 (PMC11045653; doi:10.1007/s10552-023-01842-4)
Supplement: Supplementary file 2 — Supplementary file2 (EPS 56 kb) Online Resource F2. Distribution of causes of death by stage at diagnosis, overall (“final estimate”) and by computational step, including observation (“known deaths”), imputation due to loss to follow-up (“lost imputed deaths”), or extrapolation due to survival beyond the end of follow-up (“extrapolated deaths”). As shown, at stages I and II, 40% of causes of death were known from observation, 5% were imputed, and 55% were extrapolated; at stage III, 68% of causes of death were known from observation, 3% were imputed, and 29% were extrapolated; and at stage IV, 91% of causes of death were known from observation, 2% were imputed, and 8% were extrapolated. Index cancer deaths are likely to occur relatively soon after diagnosis, whereas other deaths are likely to occur later (often beyond 5 years after diagnosis). As shown, the time dependency in observed causes of death differs by stage at diagnosis, and is accounted for through extrapolation. [file 10552_2023_1842_MOESM2_ESM.pdf]

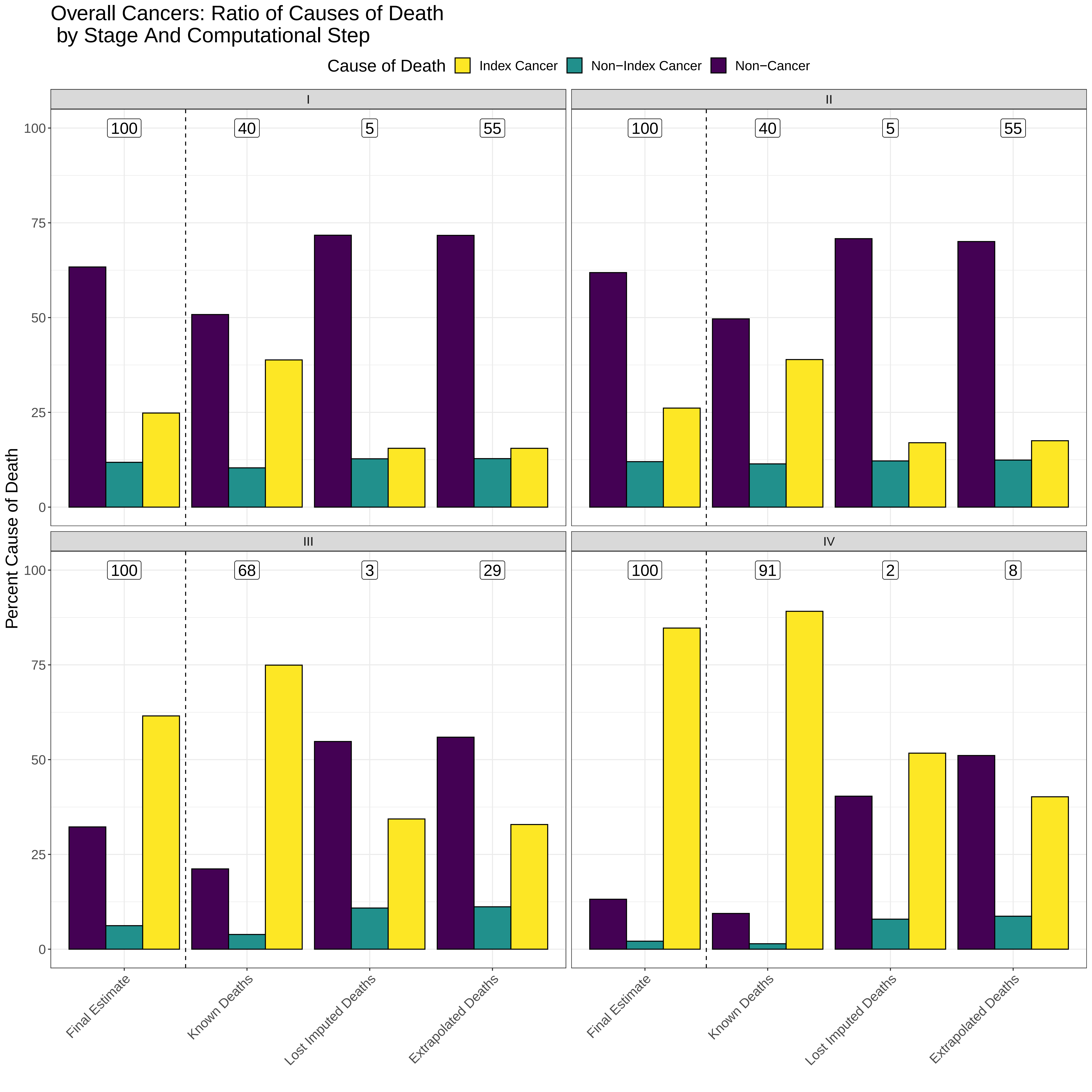

Overall Cancers: Ratio of Causes of Death
by Stage And Computational Step
Cause of Death
Index Cancer
Non−Index Cancer
Non−Cancer
I
II
III
IV
100
40
5
55
100
40
5
55
100
68
3
29
100
91
2
8
Percent Cause of Death
0
25
50
75
100
Final Estimate
Known Deaths
Lost Imputed Deaths
Extrapolated Deaths
