# Supplementary figures and images for: Avoiding lead-time bias by estimating stage-specific proportions of cancer and non-cancer deaths
Source: Cancer Causes Control. 2024 Jan 18;35(5):849–64. doi: 10.1007/s10552-023-01842-4 (PMC11045653; doi:10.1007/s10552-023-01842-4)

Cause of Death: Non-Index Cancers by Stage

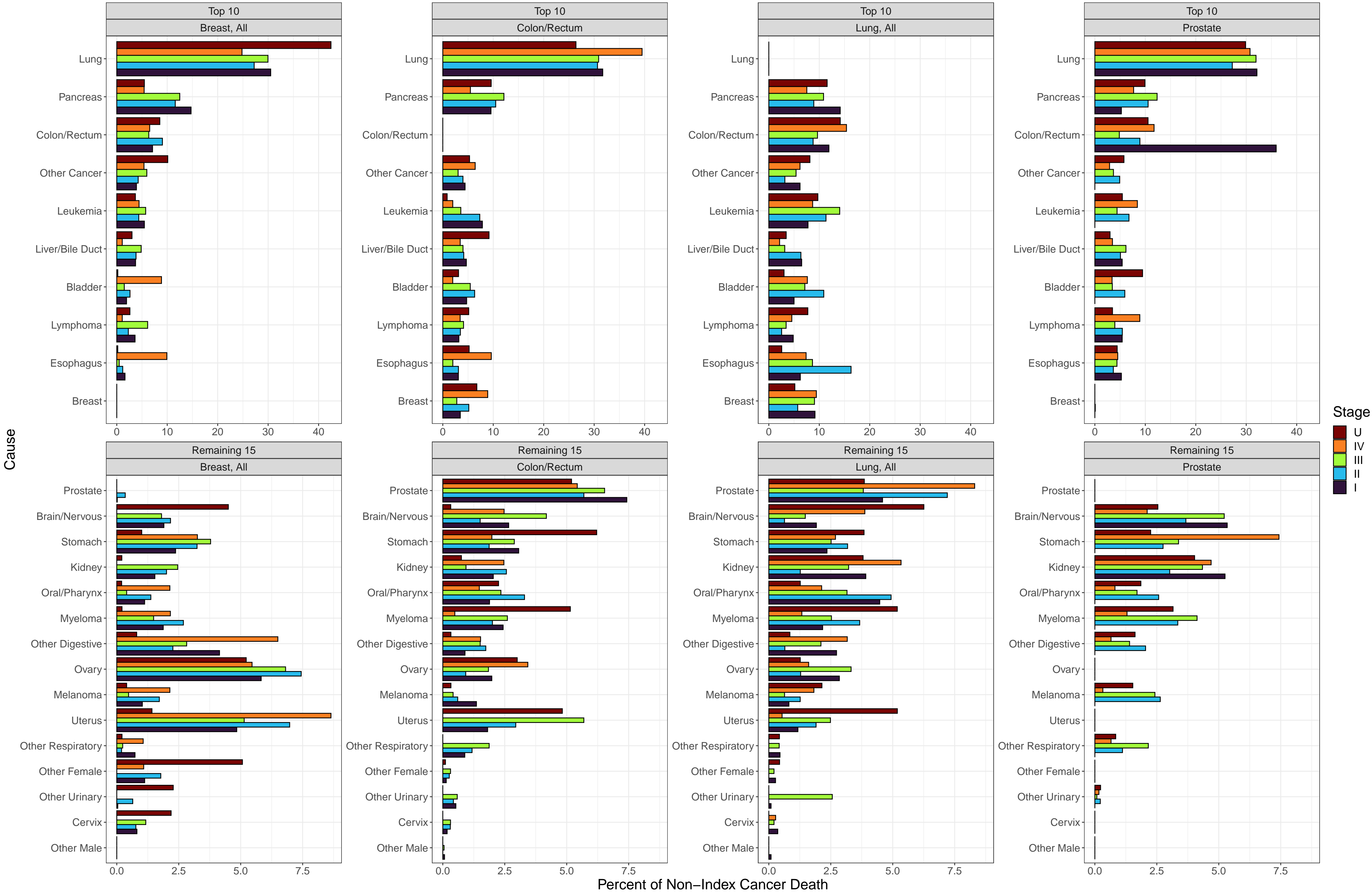

Supplement: Supplementary file 3 — Supplementary file3 (EPS 55 kb) Online Resource F3. Distribution of detailed non-index cancer causes of death (extrapolated if not observed) by stage at diagnosis for cases with primary index cancer of the breast, colon/rectum, lung, and prostate, ages 50–84 years at diagnosis from 2006–2010, followed for mortality through 2020, Surveillance, Epidemiology, and End Results (SEER) 17 registries. U: unknown/missing stage. [file 10552_2023_1842_MOESM3_ESM.pdf]

Cause of Death: Non-Cancers by Stage

Cause

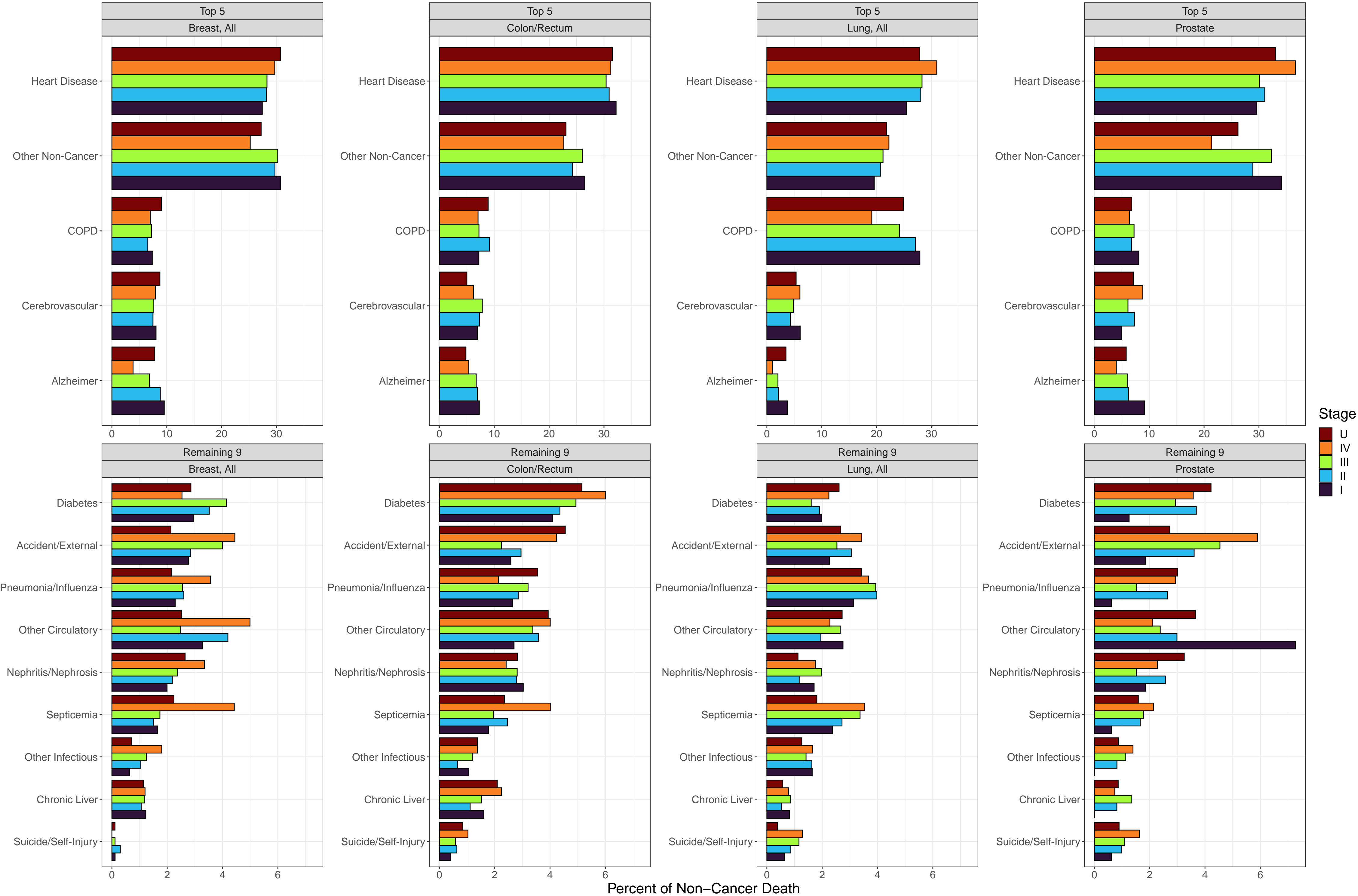

Stage

U

IV

III

II

I

Percent of Non-Cancer Death

Supplement: Supplementary file 4 — Supplementary file4 (EPS 44 kb) Online Resource F4. Distribution of detailed non-cancer causes of death (extrapolated if not observed) by stage at diagnosis for cases with primary index cancer of the breast, colon/rectum, lung, and prostate, ages 50–84 years at diagnosis from 2006–2010, followed for mortality through 2020, Surveillance, Epidemiology, and End Results (SEER) 17 registries. COPD: chronic obstructive pulmonary disease; U: unknown/missing stage. [file 10552_2023_1842_MOESM4_ESM.pdf]

# Cause of Death By Age and Stage

Cause of Death   Index Cancer   Non-Index Cancer   Non-Cancer

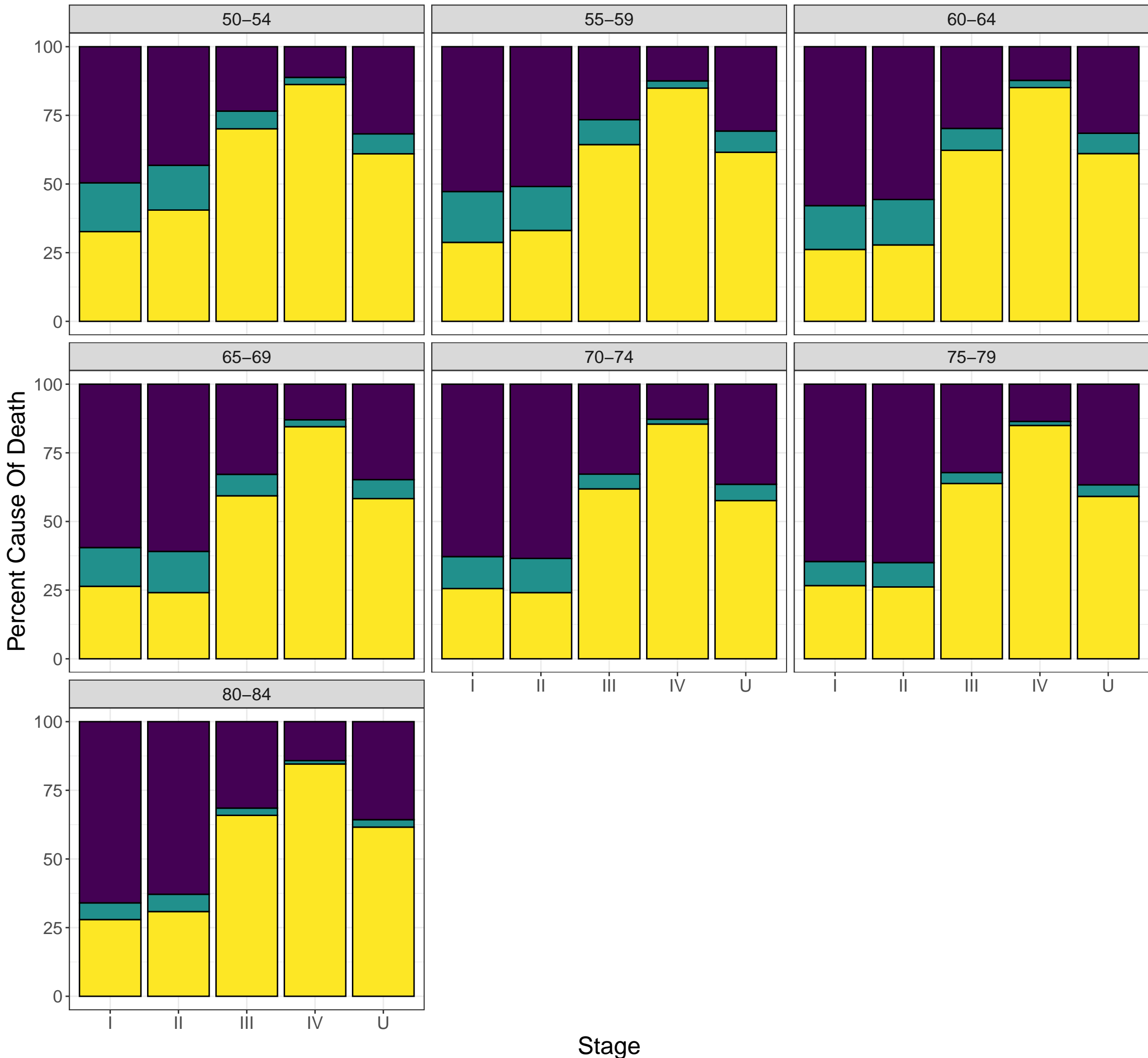

Supplement: Supplementary file 5 — Supplementary file5 (EPS 27 kb) Online Resource F5. Age-stratified distribution of causes of death (extrapolated if not observed) by stage at diagnosis for cancer cases of all types combined, ages 50–84 years at diagnosis from 2006–2010, followed for mortality through 2020, Surveillance, Epidemiology, and End Results (SEER) 17 registries. U: unknown/missing stage. [file 10552_2023_1842_MOESM5_ESM.pdf]

# Cause of Death By Sex and Stage

Cause of Death   Index Cancer   Non-Index Cancer   Non-Cancer

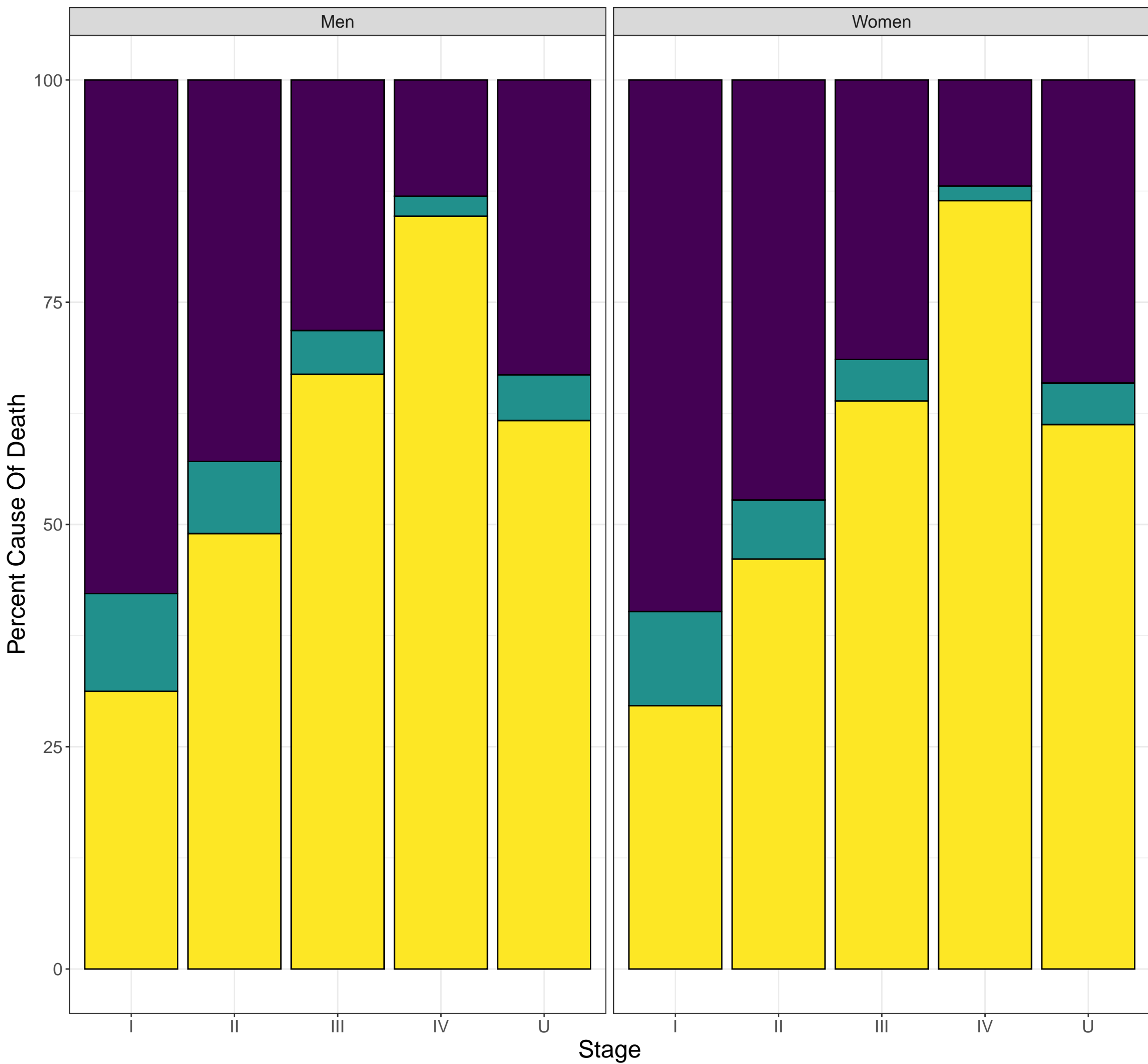

Supplement: Supplementary file 6 — Supplementary file6 (EPS 13 kb) Online Resource F6. Sex-stratified distribution of causes of death (extrapolated if not observed) by stage at diagnosis for cancer cases of all types combined, excluding breast cancer, female genital cancers, and male genital cancers, ages 50–84 years at diagnosis from 2006–2010, followed for mortality through 2020, Surveillance, Epidemiology, and End Results (SEER) 17 registries. U: unknown/missing stage. [file 10552_2023_1842_MOESM6_ESM.pdf]
