# Supplementary material for: Avoiding lead-time bias by estimating stage-specific proportions of cancer and non-cancer deaths
Source: Cancer Causes Control. 2024 Jan 18;35(5):849–64. doi: 10.1007/s10552-023-01842-4 (PMC11045653; doi:10.1007/s10552-023-01842-4)
Supplement: Supplementary file 7 — Supplementary file7 (DOCX 23 kb) [file 10552_2023_1842_MOESM7_ESM.docx]

**Online Resource T1. Stage-specific distribution of detailed non-index cancer and non-cancer causes of death (extrapolated if not observed) among cancer cases of all types combined, ages 50–84 years at diagnosis in 2006–2010, followed for mortality through 2020, Surveillance, Epidemiology, and End Results (SEER) 17 registries.** Causes of death are ordered by frequency for stage I index cancer. Column percentages shown are within categories of non-index cancer and non-cancer causes of death, and may not sum to 100% due to rounding error.

|  |  | Stage I | | Stage II | | Stage III | | Stage IV | | Unknown/Missing Stage | |
| --- | --- | --- | --- | --- | --- | --- | --- | --- | --- | --- | --- |
|  |  | *n* | % | *n* | % | *n* | % | *n* | % | *n* | % |
| Non-Index Cancer | |  |  |  |  |  |  |  |  |  |  |
|  | Lung | 8,417 | 26.5% | 10,993 | 28.1% | 2,679 | 28.3% | 1,268 | 29.6% | 3,559 | 31.3% |
|  | Pancreas | 4,028 | 12.7% | 4,100 | 10.5% | 1,106 | 11.7% | 348 | 8.1% | 1,018 | 9.0% |
|  | Colon/Rectum | 2,360 | 7.4% | 3,157 | 8.1% | 478 | 5.0% | 399 | 9.3% | 910 | 8.0% |
|  | Leukemia | 1,529 | 4.8% | 2,443 | 6.2% | 487 | 5.1% | 166 | 3.9% | 465 | 4.1% |
|  | Other Cancer | 1,477 | 4.6% | 1,839 | 4.7% | 468 | 4.9% | 213 | 5.0% | 726 | 6.4% |
|  | Breast | 1,424 | 4.5% | 335 | 0.9% | 275 | 2.9% | 189 | 4.4% | 403 | 3.5% |
|  | Liver/Intrahepatic Bile Duct | 1,418 | 4.5% | 1,948 | 5.0% | 468 | 4.9% | 182 | 4.3% | 500 | 4.4% |
|  | Lymphoma | 1,358 | 4.3% | 1,794 | 4.6% | 383 | 4.0% | 123 | 2.9% | 318 | 2.8% |
|  | Prostate | 1,300 | 4.1% | 406 | 1.0% | 267 | 2.8% | 197 | 4.6% | 398 | 3.5% |
|  | Ovary | 948 | 3.0% | 427 | 1.1% | 196 | 2.1% | 47 | 1.1% | 243 | 2.1% |
|  | Brain/Other Nervous | 945 | 3.0% | 1,198 | 3.1% | 301 | 3.2% | 100 | 2.3% | 287 | 2.5% |
|  | Other Digestive | 855 | 2.7% | 784 | 2.0% | 163 | 1.7% | 71 | 1.7% | 225 | 2.0% |
|  | Bladder | 822 | 2.6% | 2,149 | 5.5% | 380 | 4.0% | 156 | 3.6% | 514 | 4.5% |
|  | Myeloma | 795 | 2.5% | 1,173 | 3.0% | 220 | 2.3% | 38 | 0.9% | 168 | 1.5% |
|  | Stomach | 751 | 2.4% | 1,042 | 2.7% | 280 | 3.0% | 130 | 3.0% | 310 | 2.7% |
|  | Esophagus | 740 | 2.3% | 1,286 | 3.3% | 346 | 3.7% | 229 | 5.3% | 349 | 3.1% |
|  | Uterus | 644 | 2.0% | 443 | 1.1% | 231 | 2.4% | 47 | 1.1% | 136 | 1.2% |
|  | Oral Cavity/Pharynx | 570 | 1.8% | 1,075 | 2.7% | 218 | 2.3% | 123 | 2.9% | 196 | 1.7% |
|  | Kidney | 560 | 1.8% | 1,074 | 2.7% | 220 | 2.3% | 106 | 2.5% | 290 | 2.5% |
|  | Melanoma | 320 | 1.0% | 860 | 2.2% | 113 | 1.2% | 87 | 2.0% | 186 | 1.6% |
|  | Other Respiratory | 192 | 0.6% | 361 | 0.9% | 96 | 1.0% | 50 | 1.2% | 65 | 0.6% |
|  | Other Female | 144 | 0.5% | 88 | 0.2% | 26 | 0.3% | 3 | 0.1% | 55 | 0.5% |
|  | Cervix | 108 | 0.3% | 45 | 0.1% | 24 | 0.3% | 3 | 0.1% | 23 | 0.2% |
|  | Other Urinary | 57 | 0.2% | 103 | 0.3% | 43 | 0.5% | 1 | 0.0% | 19 | 0.2% |
|  | Other Male | 22 | 0.1% | 2 | 0.0% | 2 | 0.0% | 3 | 0.1% | 14 | 0.1% |
| Non-Cancer | |  |  |  |  |  |  |  |  |  |  |
|  | Heart Disease | 50,265 | 29.5% | 61,601 | 30.5% | 14,669 | 29.8% | 8,410 | 31.5% | 22,774 | 30.9% |
|  | Other Non-Cancer | 47,696 | 28.0% | 56,808 | 28.2% | 13,350 | 27.1% | 6,459 | 24.2% | 19,376 | 26.3% |
|  | Chronic Obstructive Pulmonary Disease | 15,616 | 9.2% | 14,794 | 7.3% | 4,530 | 9.2% | 2,648 | 9.9% | 5,999 | 8.1% |
|  | Cerebrovascular | 12,340 | 7.2% | 14,737 | 7.3% | 3,537 | 7.2% | 1,813 | 6.8% | 5,006 | 6.8% |
|  | Alzheimer | 12,140 | 7.1% | 12,999 | 6.4% | 2,651 | 5.4% | 910 | 3.4% | 3,443 | 4.7% |
|  | Diabetes | 5,972 | 3.5% | 7,658 | 3.8% | 1,792 | 3.6% | 885 | 3.3% | 2,991 | 4.1% |
|  | Other Circulatory | 5,148 | 3.0% | 6,411 | 3.2% | 1,538 | 3.1% | 767 | 2.9% | 2,197 | 3.0% |
|  | Accident/External | 5,146 | 3.0% | 6,971 | 3.5% | 1,572 | 3.2% | 1,176 | 4.4% | 2,517 | 3.4% |
|  | Pneumonia/Influenza | 4,540 | 2.7% | 5,489 | 2.7% | 1,421 | 2.9% | 1,007 | 3.8% | 2,398 | 3.3% |
|  | Septicemia | 3,454 | 2.0% | 3,602 | 1.8% | 1,173 | 2.4% | 851 | 3.2% | 1,794 | 2.4% |
|  | Other Infectious | 2,011 | 1.2% | 1,997 | 1.0% | 772 | 1.6% | 500 | 1.9% | 1,345 | 1.8% |
|  | Nephritis/Nephrosis | 3,377 | 2.0% | 4,981 | 2.5% | 1,090 | 2.2% | 607 | 2.3% | 2,291 | 3.1% |
|  | Chronic Liver | 1,945 | 1.1% | 1,908 | 0.9% | 680 | 1.4% | 373 | 1.4% | 1,031 | 1.4% |
|  | Suicide/Self-Injury | 670 | 0.4% | 1,764 | 0.9% | 408 | 0.8% | 293 | 1.1% | 486 | 0.7% |

**Online Resource T2. Stage-specific distribution of causes of death (extrapolated if not observed) among cancer cases of all types combined by race/ethnicity, ages 50–84 years at diagnosis in 2006–2010, followed for mortality through 2020, Surveillance, Epidemiology, and End Results (SEER) 17 registries.** Row percentages may not sum to 100% due to rounding error.

|  |  | Overall Cause of Death | | | | |  |
| --- | --- | --- | --- | --- | --- | --- | --- |
|  |  | Index Cancer | | Non-Index Cancer | | Non-Cancer | |
| Race/Ethnicity | Stage at Diagnosis | *n* | % | *n* | % | *n* | % |
| White, Non-Hispanic | |  |  |  |  |  |  |
|  | I | 48,986 | 24% | 24,838 | 12% | 133,332 | 64% |
|  | II | 60,103 | 26% | 28,600 | 12% | 145,430 | 62% |
|  | III | 67,244 | 61% | 6,941 | 6% | 36,178 | 33% |
|  | IV | 123,062 | 84% | 3,119 | 2% | 19,529 | 13% |
|  | Unknown/Missing | 85,143 | 58% | 8,102 | 6% | 52,678 | 36% |
| Black, Non-Hispanic | |  |  |  |  |  |  |
|  | I | 6,128 | 32% | 2,057 | 11% | 11,147 | 58% |
|  | II | 10,004 | 25% | 4,460 | 11% | 24,862 | 63% |
|  | III | 10,457 | 66% | 805 | 5% | 4,628 | 29% |
|  | IV | 19,608 | 86% | 444 | 2% | 2,858 | 12% |
|  | Unknown/Missing | 11,787 | 58% | 1,198 | 6% | 7,400 | 36% |
| Hispanic |  |  |  |  |  |  |  |
|  | I | 5,871 | 27% | 2,452 | 11% | 13,535 | 62% |
|  | II | 8,803 | 29% | 3,359 | 11% | 17,702 | 59% |
|  | III | 8,692 | 61% | 911 | 6% | 4,646 | 33% |
|  | IV | 15,836 | 85% | 453 | 2% | 2,449 | 13% |
|  | Unknown/Missing | 13,151 | 61% | 1,105 | 5% | 7,303 | 34% |
| Asian American/Pacific Islander, Non-Hispanic | | |  |  |  |  |  |
|  | I | 5,126 | 30% | 2,083 | 12% | 10,010 | 58% |
|  | II | 5,564 | 30% | 2,340 | 12% | 10,837 | 58% |
|  | III | 6,764 | 63% | 767 | 7% | 3,236 | 30% |
|  | IV | 11,901 | 87% | 227 | 2% | 1,596 | 12% |
|  | Unknown/Missing | 7,991 | 62% | 702 | 5% | 4,299 | 33% |
| American Indian/Alaska Native, Non-Hispanic | | |  |  |  |  |  |
|  | I | 417 | 32% | 153 | 12% | 728 | 56% |
|  | II | 418 | 29% | 137 | 9% | 909 | 62% |
|  | III | 602 | 64% | 46 | 5% | 288 | 31% |
|  | IV | 1,079 | 86% | 15 | 1% | 161 | 13% |
|  | Unknown/Missing | 746 | 63% | 50 | 4% | 388 | 33% |
